# Cell force-mediated matrix reorganization underlies multicellular network assembly

**DOI:** 10.1038/s41598-018-37044-1

**Published:** 2019-01-09

**Authors:** Christopher D. Davidson, William Y. Wang, Ina Zaimi, Danica Kristen P. Jayco, Brendon M. Baker

**Affiliations:** Department of Biomedical Engineering, University of Michigan, Ann Arbor, MI 48109 USA

## Abstract

Vasculogenesis is the *de novo* formation of a vascular network from individual endothelial progenitor cells occurring during embryonic development, organogenesis, and adult neovascularization. Vasculogenesis can be mimicked and studied *in vitro* using network formation assays, in which endothelial cells (ECs) spontaneously form capillary-like structures when seeded in the appropriate microenvironment. While the biochemical regulators of network formation have been well studied using these assays, the role of mechanical and topographical properties of the extracellular matrix (ECM) is less understood. Here, we utilized both natural and synthetic fibrous materials to better understand how physical attributes of the ECM influence the assembly of EC networks. Our results reveal that active cell-mediated matrix recruitment through actomyosin force generation occurs concurrently with network formation on Matrigel, a reconstituted basement membrane matrix regularly used to promote EC networks, and on synthetic matrices composed of electrospun dextran methacrylate (DexMA) fibers. Furthermore, modulating physical attributes of DexMA matrices that impair matrix recruitment consequently inhibited the formation of cellular networks. These results suggest an iterative process in which dynamic cell-induced changes to the physical microenvironment reciprocally modulate cell behavior to guide the formation and stabilization of multicellular networks.

## Introduction

Vasculogenesis, the *de novo* formation of blood vessels, occurs during embryonic development, organogenesis, and adult neovascularization^1–3^. This dynamic process involves the aggregation and organization of individual endothelial progenitor cells into an interconnected network of capillaries^4^. Due to numerous challenges studying vasculogenesis *in vivo*, *in vitro* network formation assays have greatly facilitated our understanding of the biological regulation of this complex process. In typical studies, endothelial cells (ECs) plated on Matrigel (a reconstituted gel containing basement membrane matrix proteins) rapidly attach, extend, and form networks of multicellular capillary-like tubules within 24 hours^5^. These and similar studies performed with two- or three-dimensional collagen and fibrin gels^6,7^ have been critical in determining the vital growth factors^8^, genes^9^, and signaling pathways^10^ required for vasculogenesis, but far less is known about how physical attributes of the extracellular matrix (ECM) govern this cell assembly process. A deeper understanding from the perspective of the physical microenvironment would aid in the design of biomaterials that facilitate the rapid formation of vasculature and subsequent host integration following implantation, which are significant outstanding challenges in the field of tissue engineering and regenerative medicine^11^.

Mechanical interactions between cells and the ECM are critical in many single- and multi-cellular processes including cell spreading^12^, cell migration^13^, and tissue morphogenesis^14^. Previous work implicating matrix mechanical properties in vasculogenesis has focused on matrix elastic modulus and generally suggests that the more compliant a material is, the greater its capacity to facilitate EC network formation^15^. For example, Vailhé *et al*. varied fibrin gel concentration and resulting elastic modulus, and observed that decreasing fibrin concentrations led to an increase in EC network formation^6^. However, given the co-dependence of gel mechanical properties, matrix topography, and ligand density on ECM protein concentration within naturally derived gels, isolating the specific contribution of biophysical vs. biochemical signals proves difficult. One approach to counteract this problem in natural materials is glycation, which increases gel elastic modulus with minimal impact on gel architecture^16^. Using this technique in collagen gels, Mason *et al*. found that increasing ECM stiffness correlated with the formation of capillary-like structures, a result opposite to the accepted trend^17^. Synthetic ECM mimetics typically provide greater control over matrix cues and therefore have provided another key approach to examining the role of matrix mechanical properties on EC network formation. Studies conducted on polyacrylamide (PA) gels, a commonly used elastic hydrogel that enables independent modulation of ECM elastic modulus and ligand density, conversely demonstrated that ECs transition from a monolayer to network-like phenotype with decreasing hydrogel elastic modulus^18–20^.

One potential source of these conflicting observations is that elastic modulus, although an important metric representing the stress/strain response of an elastically deforming material, may not sufficiently describe the mechanical behavior of all biologic materials. *In vivo* microenvironments that promote vessel formation, such as embryonic mesenchymal tissue during development or fibrin-clots during wound healing, possess complex mechanical behavior due in part to their fibrous composition and viscoelastic properties. Indeed, many of the settings commonly used to promote the formation of EC networks *in vitro* – Matrigel, collagen, and fibrin – also possess fibrous structure at various length scales with complex and hierarchical mechanics not fully encapsulated by an elastic modulus value^21–24^.

In particular, we recently showed that in fibrous matrices, cellular ECM mechanosensing is affected by dynamic changes in local adhesive ligand availability and matrix topography due to cell-force mediated recruitment of matrix fibers^25^. Cellular reorganization of the matrix has also been observed *in vivo*^26^, in natural materials such as collagen and fibrin^27,28^, and within stress relaxing hydrogels^29^. Interestingly, matrix reorganization in many of these materials has been shown to be irreversible, implying plastic deformation that permanently alters matrix architecture^24,28^. Elastic hydrogels such as PA, however, support limited matrix reorganization^25,30^, and any deformation to the underlying substrate under cell forces is completely reversible (a behavior essential to the use of elastic materials for traction force methodologies)^31^. As the majority of EC network formation studies in synthetic ECMs have focused on non-fibrous elastic hydrogels, the relationship between matrix reorganization and vasculogenesis has not been explored. Here, we combined experiments in natural and synthetic materials to gain insight into how physical properties of fibrous ECM and cell-mediated matrix reorganization regulate network formation. We established a model of EC network formation in a synthetic fibrous matrix, orthogonally examined the effect of matrix architecture and mechanics on this assembly process, and found that cell-force mediated matrix reorganization and continued force propagation is required for the formation and stabilization of these networks.

## Results

### ECM mechanics regulate EC network formation and matrix reorganization on Matrigel

**Figure 1:**
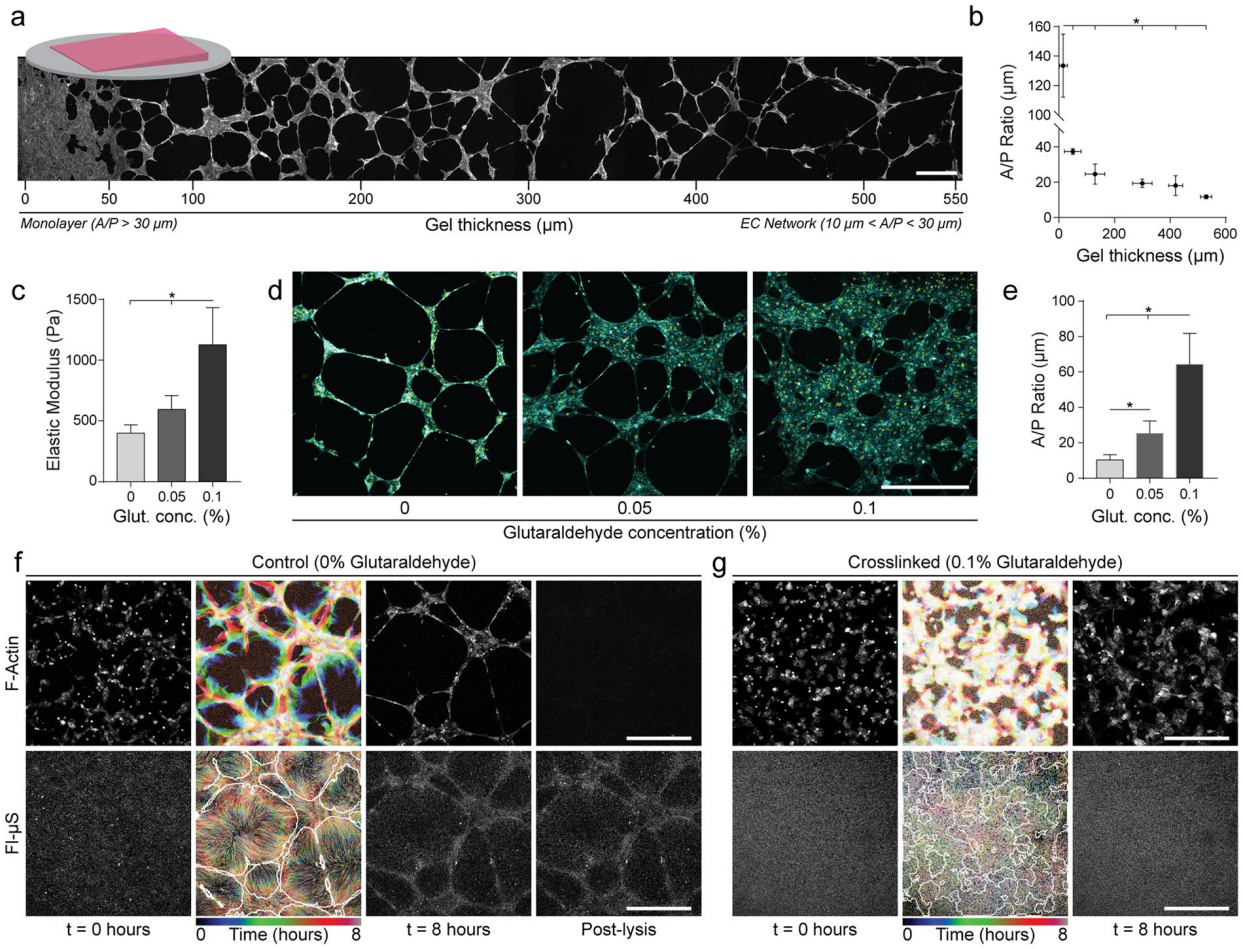
ECM mechanics regulate EC network formation and matrix reorganization on Matrigel. (**a**) Confocal fluorescence maximum projections of phalloidin-stained ECs seeded on wedge-shaped Matrigel substrates ranging in thickness from approximately 0 to 550 µm, after 12 h of culture. (**b**) Resulting network morphology was determined by the area/perimeter ratio of thresholded images at select thicknesses of Matrigel as indicated (Fig. S1). Horizontal error bars represent the range of thickness for each analyzed section of the image; n ≥ 3. (**c**) Young’s modulus of Matrigel crosslinked with varying glutaraldehyde concentrations determined by microscale compression testing; n ≥ 8. (**d**) Fluorescent images of ECs cultured for 12 hours on Matrigel after glutaraldehyde crosslinking at the indicated concentration; actin (cyan), nuclei (yellow). (**e**) Cell area/perimeter ratio on Matrigel substrates as a function of glutaraldehyde crosslinking; n ≥ 7. Representative time-lapse images of lifeAct-GFP expressing ECs (top row) and embedded fluorescent microspheres (Fl-μS, bottom row) on untreated control (**f**) and 0.1% glutaraldehyde treated (**g**) Matrigel substrates. Rainbow overlays depict cell and Fl-µS motion (with final EC structure outlined in white) over the 8 hour time-lapse. Rightmost images in (**f**) depict cell and Fl-µS images following cell lysis. Untreated control substrates demonstrate high levels of matrix deformation and reorganization in contrast to glutaraldehyde crosslinked Matrigel. In the control case, limited elastic recovery is observed after removal of the formed network. Scale bars: 500 μm. All data presented as mean ± std; *P < 0.05.

To begin to investigate the role of ECM mechanics on EC network formation we utilized Matrigel, a reconstituted basement membrane matrix known to robustly promote the formation of EC networks^5^. We adopted a technique to fabricate wedge-shaped gels with varying thickness across the substrate^32^, thus modulating cell-perceived matrix stiffness via proximity to a rigid underlying boundary condition^33^. Human umbilical vein ECs seeded on these substrates and visualized after 12 hours of culture resulted in varying multicellular morphologies as a function of gel thickness (Fig. 1a). At thicker sections of the gel (>200 microns), networks formed with long cellular extensions in contrast to thinner sections (<200 microns) where extensions were shorter and yielded a denser network or a cell monolayer at the thinnest regions examined (<20 microns). To statistically differentiate these morphological variations, we utilized a previously established metric of the ratio of cellular area to perimeter (A/P ratio)^19^. This metric numerically distinguishes three possible phenotypes: single, disconnected cells (A/P < 10 μm), an interconnected network (10 μm < A/P < 30 μm), or a cell monolayer (A/P > 30 μm) (Fig. S1). In the above experiment, A/P ratio decreased significantly with increasing Matrigel thickness, supporting the morphological transition from monolayer to network phenotype (Fig. 1b).

To provide further evidence that matrix mechanical properties mediate this assembly process, Matrigel substrates of uniform thickness (approximately 450 µm) were crosslinked with glutaraldehyde^34^. Controlled exposure to varying concentrations of glutaraldehyde increased Young’s modulus from 401 to 1126 Pa (Figs 1c and S2) and led to a graded cellular response ranging from successful network formation in untreated controls (E = 401 Pa), a heterogeneous mixture of networks with large areas containing monolayers at low concentrations of glutaraldehyde (E = 595 Pa), to a complete monolayer lacking network morphology at the highest concentration examined (E = 1126 Pa) (Fig. 1d,e). Taken together, these results obtained by two distinct methods are in agreement with the general claim that more compliant materials promote the formation of EC networks *in vitro*^6,18–20^.

To visualize cell-mediated deformations of the underlying matrix, control or glutaraldehyde-crosslinked Matrigel substrates with embedded fluorescent microspheres (Fl-μS) were imaged by time-lapse microscopy immediately after seeding with ECs. In control substrates, cells appeared to exert traction forces to actively deform and recruit the ECM concurrent with cellular reorganization and network assembly, resulting in dense regions of matrix directly beneath ECs that mirrored closely the overall pattern of assembled networks (Figs 1f, S3a, Movie S1). Furthermore, these cell-mediated deformations to the matrix were permanent, as limited elastic recovery was noted following removal of EC networks by lysis (Fig. 1f). Interestingly, we observed distinct Fl-μS dynamics in crosslinked Matrigel substrates that led to monolayer formation (E = 1126 Pa). Although non-negligible motion was observed, Fl-μS displacement paths were short, randomly directed, and did not condense, implying limited ECM recruitment during the formation of a cellular monolayer (Figs 1g, S3b, Movie S2). These studies suggest an important role for matrix reorganization in EC network formation, and may in part explain why compliant, deformable matrix settings tend to be pro-vasculogenic.

### Synthetic fibrous DexMA matrices undergo pronounced matrix reorganization during EC network formation

While Matrigel has served as an important setting for studying various biological processes including vasculogenesis, eliminating the influence of its numerous biochemical components and orthogonally modulating the biophysical properties of this material proves challenging. In the experiments above, we modulated crosslinking and quantified differences in substrate elastic modulus from bulk compression testing, however glutaraldehyde crosslinking alters biochemical and mechanical properties beyond solely the elastic modulus of the gel. Furthermore, although Matrigel possesses a fibrous ultrastructure (with fibers on the range of 70 nm in diameter)^35^, tuning mechanical and topographical features significant to EC network formation is not currently achievable with this material. Given these challenges and a putative role for fibrous structure in vasculogenesis, we adopted a previously developed synthetic fibrous ECM mimetic composed of electrospun dextran methacrylate (DexMA) fibers possessing well-defined and tunable mechanical and biochemical properties^25^.

**Figure 2:**
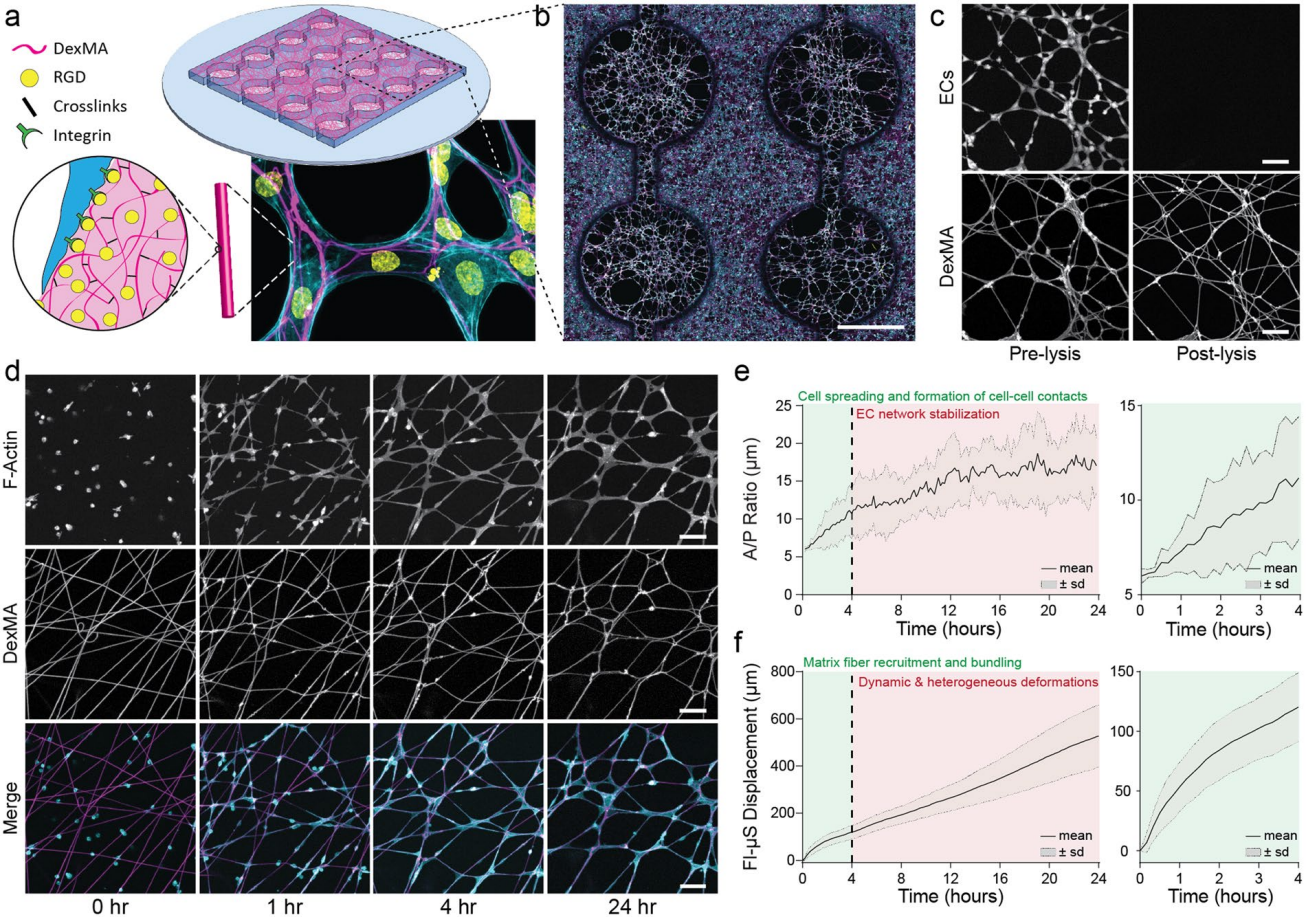
Synthetic fibrous DexMA matrices undergo pronounced matrix reorganization during EC network formation. (**a**) Schematic of microfabricated PDMS multi-well substrate possessing a 4 × 4 array of wells, each supporting a suspended matrix of non-aligned DexMA fibers coupled with RGD to facilitate cell adhesion. (**b**) Tile-scan confocal image of EC networks after 24 hours of culture on DexMA fiber matrices; rhodamine-labeled fibers (magenta), F-actin (cyan), and nuclei (yellow) (scale bar: 1 mm). (**c**) Limited elastic recovery of fibers after lysis of CellTracker labeled ECs, demonstrating that physical matrix reorganization is permanent (scale bar: 100 μm). (**d**) Representative time-lapse images of lifeAct-GFP expressing ECs at 0, 1, 4, and 24 h following seeding (scale bar: 100 μm). Cell area/perimeter ratio (**e**) and total Fl-μS displacement (**f**) over the 24-hour time-lapse series of network formation; n = 8.

DexMA fibrous matrices were suspended over an array of microfabricated wells such that cells in well-regions are not influenced by a rigid underlying support, but instead sense the mechanical properties defined by fibrous architecture, the stiffness of individual fibers, and proximity to rigid boundary conditions of the well edge (Fig. 2a). Fiber matrices with low bulk stiffness (E = 1.5 kPa) were functionalized with the adhesive peptide CCRGDS (RGD) via Michael-type addition with unreacted methacrylates, and seeded with ECs which rapidly adhered, reorganized ECM fibers, and assembled into networks within 24 hours (Fig. 2b,d, Movie S3). During the first four hours, we noted marked matrix reorganization through recruitment of fibers beneath individual ECs and lateral bundling of fibers between adhered cells, accompanied by cell spreading and the formation of cell-cell contacts (Fig. 2d, Movie S4). Following this initial phase, EC network morphology stabilized over a prolonged period of spatially and temporally heterogeneous deformations and force propagation throughout the matrix and EC network (Movie S3). Although the majority of cellular connections formed during the first four hours, additional cell extensions leading to inter-cellular connection occurred throughout the 24-hour culture period (Fig. S4). Similar to Matrigel, physical matrix reorganization of DexMA matrices proved permanent, as limited recovery of fibers to their initial geometry occurred following cell lysis (Fig. 2c). Furthermore, we note that ECs secrete fibronectin that adheres to DexMA fibers at network nodes where there is high relative fiber density (Fig. S5), which could in part explain the permanence of matrix deformations. A/P ratio throughout this dynamic process began low (<10 μm) while single cells adhered prior to spreading and connecting with other cells, rapidly increased over the first four hours as interconnected network formed, and then settled at an intermediate value (10–30 μm) following stabilization (Fig. 2e). To quantify matrix reorganization over time, Fl-μS were embedded within DexMA fibers and tracked during network formation^36^. The sum of the mean squared displacements between frames for each individual Fl-μS was calculated via custom Matlab script (Figs 2f, S6). Bead displacement increased rapidly over the first four hours paralleling the dynamics of A/P ratio, but then transitioned to a constant linear increase reflecting continuous matrix deformations and force propagation following EC network stabilization (Fig. 2f, Movie S3). We further quantified matrix remodeling by performing image analysis on the open space, or pores, between DexMA fibers (Fig. S7). Through this quantification, we observe that average pore size increases and the total number of pores decreases over time, supporting the observation that ECs bundle and condense matrix fibrils as they spread and interconnect into a multicellular assembly. Overall, these studies established EC network formation in fully synthetic DexMA fibrous matrices that, despite significant distinctions in matrix structure and adhesive ligand, reveal similarities in terms of network morphology and matrix reorganization as studies performed with Matrigel.

### Actomyosin contractility is required for EC network formation on fibrous DexMA matrices

**Figure 3:**
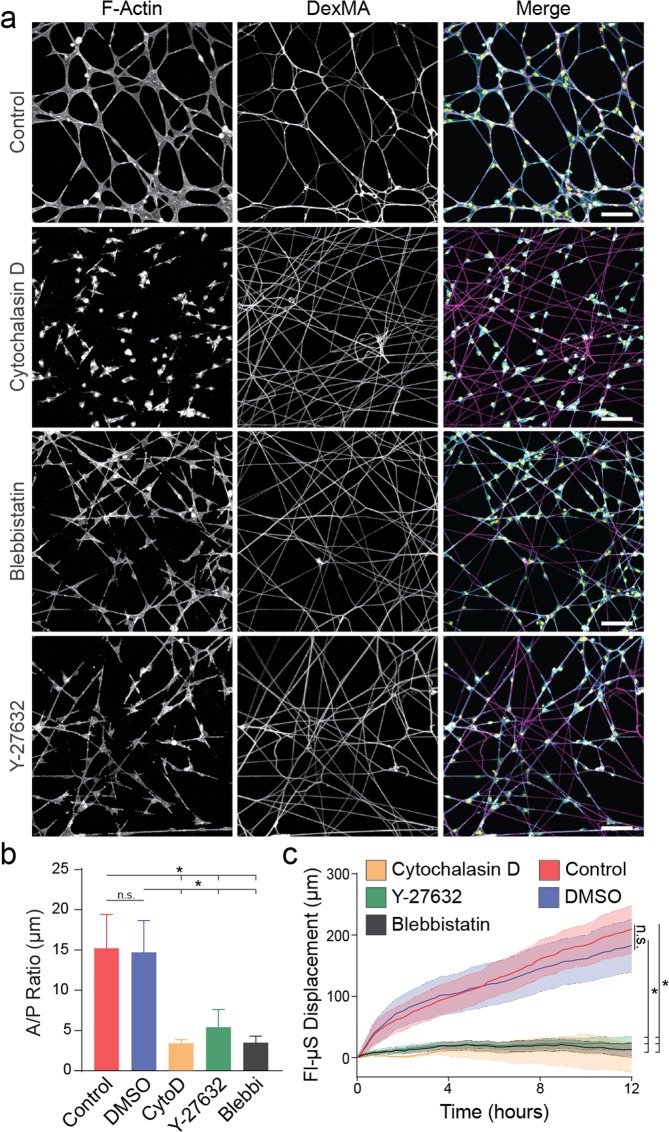
Actomyosin contractility is required for EC network formation on fibrous DexMA matrices. (**a**) Confocal fluorescence images of phalloidin-stained ECs and rhodamine-labeled fibers after 24 hours, with indicated pharmacologic inhibitor treatment; actin (cyan), nuclei (yellow), fibers (magenta). (**b**) Cell area/perimeter ratio after 24 hours of culture as a function of pharmacologic inhibitor treatment. (**c**) Total Fl-μS displacement over the first 12 hours of network formation as a function of pharmacologic inhibitor treatment. Scale bars: 100 μm. All data presented as mean ± std; n ≥ 6; *P < 0.05.

Given the likelihood that the deformations observed in the above studies are consequences of cell traction forces, we tested a requirement for actomyosin-generated contractile forces in EC network assembly by treatment with pharmacologic inhibitors. In samples dosed with cytochalasin D (actin polymerization inhibitor), blebbistatin (myosin II inhibitor), or Y-27632 (ROCK inhibitor), EC networks failed to form over 24 h (Fig. 3a). Treatment with cytochalasin D completely abrogated cell spreading, resulting in individual cells with a low A/P ratio (Fig. 3a,b). In the presence of blebbistatin and Y-27632, ECs were adhered to the matrix and spread but demonstrated limited interaction with neighboring cells and largely remained as individual cells, resulting in a lower A/P ratio as compared to controls (Fig. 3a,b). Fl-μS displacements were significantly lower for all inhibitors, corresponding to limited reorganization of matrix fibrils (Fig. 3a,c). Additional analysis of cytoskeletal structure via high-resolution confocal microscopy indicates the presence of F-actin stress fibers in control conditions, in contrast to a diffuse or punctate F-actin signal and absence of stress fibers upon treatment with cytochalasin D, blebbistatin, and Y-27632 (Fig. S8). These studies demonstrate that actomyosin-generated contractile cell forces are required for matrix reorganization, and associate the absence of matrix reorganization with failure of ECs to form networks.

### Physical properties of fibrous ECM influence matrix reorganization and EC network formation

**Figure 4:**
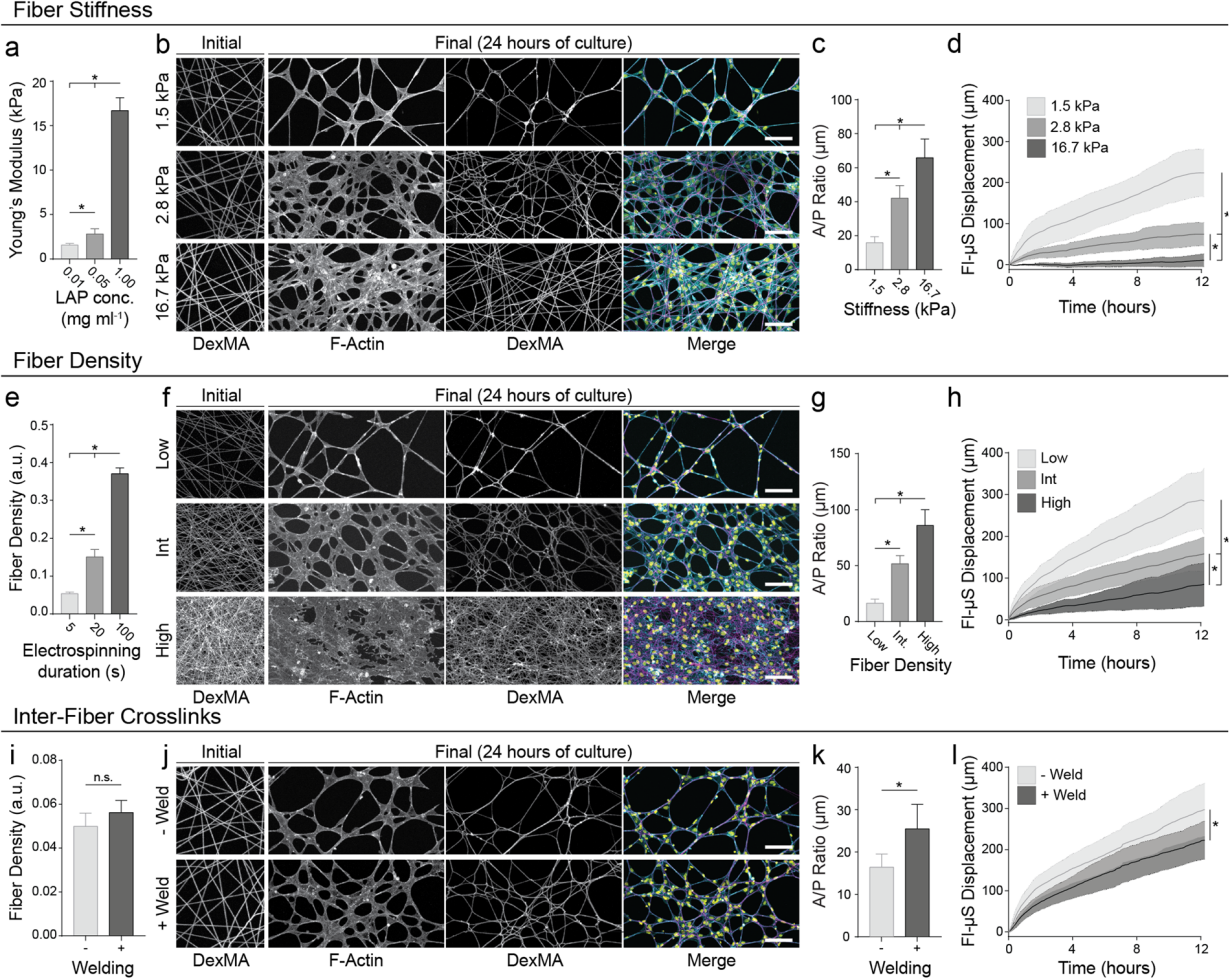
Physical properties of fibrous ECM influence matrix reorganization and EC network formation. EC network formation was assayed on DexMA fibrous matrices with varying stiffness (**a**–**d**), fiber density (**e**–**h**), and with modulation of inter-fiber crosslinks (welds) (**i**–**l**). (**a**) Young’s modulus of DexMA fiber matrices as a function of photoinitiator (LAP) concentration. (**e,i**) Fiber density prior to cell seeding determined by average pixel intensity of fluorescent fiber images. (**b,f,j**) Representative image of initial fiber density prior to cell seeding (far left); confocal fluorescent images of phalloidin-stained ECs (left) and rhodamine-labeled fibers (middle) 24 hours after seeding under the indicated matrix perturbation after 24 hours of culture; merged images (right) showing actin (cyan), nuclei (yellow), and fibers (magenta). (**c,g,k**) EC area/perimeter ratio as a function of ECM perturbation. (**d,h,l**) Total Fl-μS displacement over the first 12 hours of network formation as a function of ECM perturbation. Scale bars: 100 μm. All data presented as mean ± std; n ≥ 6; *P < 0.05.

The above results suggest a potential requirement for cell-mediated matrix reorganization during EC network formation, so we further hypothesized that matrix mechanics and architecture could regulate this process by impacting the ability of cell forces to reorganize the matrix. Taking advantage of the tunable nature of these synthetic matrices, we orthogonally modulated physical matrix properties relevant to natural tissues and examined matrix reorganization and network formation. First, the stiffness of matrix fibers was modulated by tuning crosslinking via photoinitiator concentration, resulting in increases in the Young’s moduli of fiber matrices with no effect on initial fiber density (Figs 4a,b, S2). In comparison to controls (E = 1.5 kPa) where high levels of matrix reorganization corresponded with EC network formation, matrices with increasingly stiffer fibers led to a graded decrease in network formation and a transition towards monolayer formation (Fig. 4b, Movie S5). Increases in A/P ratio as a function of fiber/matrix stiffness corroborated this change in morphology (Fig. 4c). Increasing fiber/matrix stiffness also yielded a graded decrease in matrix reorganization as determined by Fl-μS displacements (Fig. 4d).

We next investigated the effect of matrix fiber density by altering the duration of electrospinning and fiber collection (Fig. 4e,f), while maintaining a constant degree of crosslinking (equivalent to the lowest stiffness concentration). Control matrices with the lowest fiber density examined resulted in formation of EC networks, concurrent with high levels of matrix remodeling (Fig. 4f). Increasing fiber density resulted in a graded response in A/P ratio and Fl-μS displacement similar to the above studies modulating fiber stiffness (Fig. 4f–h), further supporting a strong connection between matrix reorganization and successful network formation.

Lastly, we welded junctions between fibers via exposing substrates to high humidity prior to crosslinking in order to model inter-fiber crosslinking. This perturbation does not alter overall matrix architecture, the elasticity of individual fibers, or the Young’s modulus of the bulk material, but has previously been shown to decrease fiber recruitment by mesenchymal stem cells^25^. Control conditions with limited inter-fiber welding formed networks concurrent with high levels of matrix reorganization, as in earlier studies (Fig. 4j). However, inter-fiber welding of matrices of the same crosslinking and initial fiber density (Fig. 4i,j) led to a significant increase in A/P ratio and a significant decrease in Fl-μS displacement, indicating a transition to a cellular monolayer and a decrease in matrix remodeling (Fig. 4j–l). While networks still formed in both conditions, there was a clear change in network morphology, as welding led to larger network nodes containing monolayers as compared to the control condition, similar to Matrigel substrates exposed to low concentrations of glutaraldehyde (Fig. 1d). For all matrix perturbations (stiffness, density, and inter-fiber crosslinking) described above, there were no significant differences in fiber diameter (Fig. S9).

### Matrices permissive to physical reorganization and persistent deformations yield EC networks stabilized by VE-cadherin enriched cell-cell junctions

**Figure 5:**
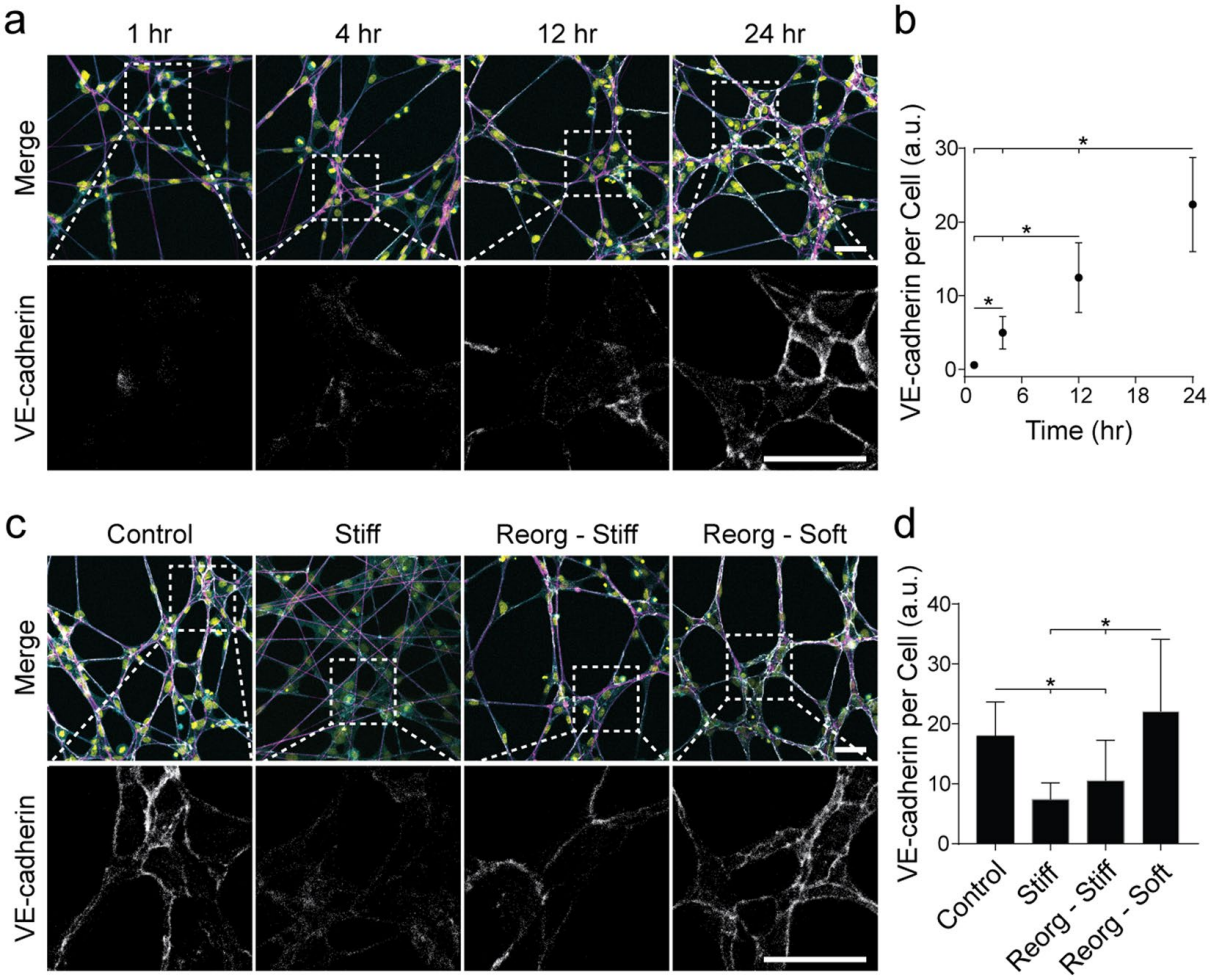
Matrices permissive to physical reorganization and persistent deformations yield EC networks stabilized by VE-cadherin enriched cell-cell junctions. (**a**) Confocal fluorescence maximum projections of phalloidin-stained ECs (cyan), rhodamine-labeled fibers (magenta), nuclei (yellow), and VE-cadherin (gray) at 0, 1, 4, and 24 h after cell seeding on control DexMA matrices (top row). Dashed boxes indicate locations of higher magnification images depicting VE-cadherin expression at cell-cell junctions (bottom row). (**c**) Confocal fluorescence maximum projections of phalloidin-stained ECs (cyan), rhodamine-labeled fibers (magenta), nuclei (yellow), and VE-cadherin (gray) of samples 24 h after seeding on control and stiff DexMA matrices, on control matrices cultured for 24 h, lysed, crosslinked, and then reseeded for 24 h (Reorg – Stiff), or on control matrices cultured for 24 h, lysed, and then reseeded for 24 h (Reorg – Soft) (top row). Dashed boxes indicate locations of higher magnification images depicting VE-cadherin expression at cell-cell junctions 24 h after cell seeding (bottom row). (**b,d**) Quantification of total VE-cadherin fluorescent intensity normalized to cell density at each timepoint (**b**) and matrix condition (**d**). Scale bars: 50 μm. All data presented as mean ± std; n ≥ 16; *P < 0.05.

These results support a role for cell force-mediated matrix reorganization in EC network formation (primarily over the first 4 hours of culture), but do not address the stabilization of networks and ensuing steady state behavior. A constant A/P ratio with linearly increasing Fl-μS displacements after the initial four hours of culture (Fig. 2e,f) reflect stable cellular connections and network morphology despite continual deformations to the underlying matrix (Movie S3). To investigate stabilization of EC networks, we examined cell-cell adhesion maturity via immunostaining for VE-cadherin, an EC adherens junction molecule critical to blood vessel maturation and stability. Fluorescent intensity of VE-cadherin (normalized to cell density) significantly increased over time on control matrices (Fig. 5a,b). In contrast, cell-cell adhesions in stiff matrices that underwent limited reorganization possessed significantly lower VE-cadherin levels despite successful spreading and formation of cell-cell contacts (Fig. 5c,d). Furthermore, treatment of formed cell networks on control matrices with ethylene glycol-bis(2-aminoethylether)-*N,N,N’,N’*-tetraacetic acid (EGTA), which chelates calcium ions and disrupts cadherin-mediated cell-cell adhesions^37,38^, resulted in a loss of intercellular connections and dissociation into single cells (Fig. S10).

Both matrix reorganization and force transmission may contribute to the observed maturation of cell-cell adhesions, however the above experiment fails to segregate the two. Matrix reorganization to a condensed architecture with restricted fiber/ligand availability could more rapidly facilitate cell-cell engagement through contact guidance, but alternatively, a mechanically permissive matrix that can be reorganized by cells may also facilitate force transmission between interconnected cells that would strengthen cadherin junctions. To discriminate between topographical and mechanical effects, we created soft and stiff matrices with pre-organized fiber architecture reflecting the final state of control matrices in the above studies. Pre-organized matrices were fabricated by allowing networks to form normally followed by cell lysis, as matrix reorganization is largely permanent (Fig. 2c). Following lysis, pre-organized matrices were either UV crosslinked and reseeded ("Reorg – Stiff") or immediately reseeded with ECs ("Reorg – Soft"). Reseeding of reorganized stiff matrices which allow for no further matrix deformations after initial organization resulted in significantly lower VE-cadherin expression as compared to control networks, despite retaining a network-like morphology due to contact guidance of pre-organized fibers (Fig. 5c,d). In contrast, the reorganized soft matrices that allow for continued matrix displacements and force propagation resulted in cell-cell junctions with significantly higher VE-cadherin (Fig. 5c,d). Taken together, these results suggest that matrix deformability and force transmission after initial interconnections form between cells stabilize EC networks through promoting the maturation of cell-cell adhesions.

## Discussion

Mechanical interactions between cells and the ECM have been shown to be crucial in many biological processes, including migration, differentiation, and morphogenesis. Here, we utilized both natural and synthetic matrices to examine how cell interactions with the physical microenvironment mediate the assembly of multicellular networks and found that dynamic force-mediated modulation of matrix structure is critical in this phenomenon. Studies varying the rigid boundary conditions and crosslinking of Matrigel demonstrated that mechanical perturbations to the microenvironment profoundly altered this process. Interestingly, we observed that active matrix recruitment coincided with network formation, resulting in regions of condensed matrix that closely paralleled the patterning of EC networks. Next, we identified matrix conditions that facilitate rapid EC network assembly on synthetic DexMA fibrous matrices affording controllable mechanical and topographical properties. We confirmed a requirement for actyomyosin-generated forces in matrix fibril reorganization and observed a strong association between dynamic changes to matrix structure and successful EC network assembly. Varying physical parameters of these matrices, we further found that perturbations that diminish the ability of cells to physically reorganize the matrix proved inhibitory to network formation. Lastly, we found that deformable matrices that permit active force transmission across an interconnected cell network promoted stabilization of cell-cell junctions as seen by increased VE-cadherin levels. Taken together, these results demonstrate that cell force-mediated reorganization, independent of enzymatic activity, mediates dynamic changes in matrix topography, ligand availability, and mechanical forces that underlie the formation and stabilization of interconnected multicellular networks.

Highly localized reorganization of the matrix through cellular recruitment of matrix fibrils results in actively changing adhesive ligand distribution and density. Our previous work demonstrated that cell-mediated fiber recruitment increased adhesive ligand density proximal to the cell surface, contributing to increases in cell spreading, focal adhesion formation, and associated signaling^25^. Similar findings have been confirmed in natural fibrous materials, such as type I collagen gels^39^, and may occur at a smaller length scale in viscoelastic hydrogels^40^. Studies performed on 2D elastic hydrogels increasing adhesive ligand concentration while maintaining gel elastic modulus constant revealed increases in cell spreading and cell force generation as measured by traction force microscopy (TFM)^41–43^. TFM in fibrous matrices is not yet an established technique due to their composition of discrete fibers (thereby invalidating continuum assumptions), non-linear mechanical behavior, and plastic deformations, thus a direct relationship between cell-generated forces and fibril/ligand organization remains an outstanding challenge. However, given previous studies in a variety of settings connecting ligand availability with focal adhesion maturation^25,39^ and traction force generation^42,44,45^, it is likely that local matrix recruitment in this setting feeds back to increase cell force generation. This suggests the initial phase of EC network assembly involves a cyclic process in which ECs initially adhere and recruit matrix, triggering increased spreading, focal adhesion formation, and force generation, which in turn could further amplify matrix recruitment until an equilibrium is eventually achieved.

Reorganization of the matrix likely also contributes to dynamic and heterogeneous changes in matrix topography and mechanics. In isotropic matrices lacking fibril alignment, cell-generated forces generally lead to radial alignment of fibers around the cell^25,46^. However, when cells are in close proximity or sense mechanical resistance from a nearby rigid boundary, cell and force anisotropies rapidly develop^47,48^. In these studies, localized fiber alignment between two cells could contribute to directional extension and formation of cellular interconnections via contact guidance, supporting a role for matrix recruitment and fiber bundling in multicellular assembly. Beyond topography-induced contact guidance, however, the presence of aligned fibers spanning neighboring cells over large distances (up to 300 microns, Fig. S11) also suggests the involvement of long-range mechanical force propagation. Heterogeneous force distribution and localized paths of highest tension could induce cell polarization, directional extension, and bundling of matrix fibers. Subsequent alterations to local matrix structure would then reinforce a preferential direction of force transmission along tensile tracks of aligned fibers between neighboring cells. Fibrous matrices in particular have been shown to propagate and focus cell-generated force across large distances^49–52^, and previous studies have implicated cell-cell communication via force transmission through the ECM^53–55^. This notion is supported here by experiments on synthetic fibrous matrices where diminished intracellular contractility, and therefore low force generation and transmission, corresponded to the failure of network formation (Fig. 3). Further, increases in fiber stiffness, fiber density, and the addition of inter-fiber crosslinks, perturbations that would dampen force transmission through the ECM, all abrogated network formation (Fig. 4). Taken together, these experiments suggest matrix-regulated mechanical forces enable cells to communicate their position over long distances in order to assemble into networks.

The above provides potential mechanisms influencing cell extension leading to cell-cell contact, but does not address network stabilization after these contacts are formed. Soft matrices permissive to matrix reorganization and network formation yielded cell-cell contacts enriched for VE-cadherin compared to stiff matrices, despite the presence of cell-cell contacts in both conditions. Further, on pre-organized networks, cell-cell contacts and cell networks formed in both soft and stiff conditions, but significantly higher VE-cadherin was noted in pre-organized and deformable matrices (Fig. 5). Previously, Liu *et al*. used microfabricated force sensors to show that mechanical tugging forces between cells engenders cell-cell adhesion formation and maturation^56^ and other work has demonstrated the requirement for actomyosin-generated tensile forces in maturing adherens junctions^57^. Given the appearance of dynamic cell forces throughout the assembled multicellular tissue during stabilization (>4 h, Movie S3), increases in VE-cadherin at cell-cell junctions could similarly be explained by tugging forces between cells at cell-cell junctions and throughout the matrix.

Overall, this study sheds light on the complex relationship between cell-ECM interactions during EC network formation, and highlights an interplay between active and passive mechanical cues from the microenvironment. Active mechanical cues, defined as external stimuli that act directly on a cell (e.g. applied compressive forces, fluid shear), versus passive mechanical cues, defined as physical properties of the environment that cannot directly perturb a cell (e.g. stiffness, viscosity, matrix alignment)^58^, have historically been dichotomized. Here, however, passive properties of the matrix (stiffness, density, inter-fiber connections) mediate matrix reorganization to influence active mechanical cues in the form of cell-generated forces propagated through the matrix to neighboring cells. This relationship is reciprocal - active forces can reorganize the matrix, in turn modifying passive matrix properties local to the cell. Importantly, reorganization of the matrix in these studies appeared permanent (Figs 1 and 2), implying plastic deformation, a behavior of viscoplastic materials such as Matrigel, collagen, and fibrin^24,27^. Interestingly, these three materials also facilitate network formation *in vivo* and *in vitro*, further supporting a role for matrix remodeling in this process and suggesting that the permanence of these deformations could be essential. Taken together, this information is critical to the design and development of vasculogenic biomaterials. Specifically, when designing synthetic materials to support vasculogenesis, matrix physical properties that support permanent matrix reorganization and long range force transmission should be considered. While elastic modulus influences these processes, physical properties beyond stiffness, such as matrix architecture and plasticity, also require careful consideration. In accord, this study suggests fiber reinforcement of synthetic biomaterials as a means to promote both matrix reorganization and long range cell-cell communication to enable multicellular assembly processes.

## Materials and Methods

### Reagents

All reagents were purchased from Sigma Aldrich and used as received, unless otherwise stated.

### Cell culture

Human umbilical vein endothelial cells (ECs) were cultured in endothelial growth medium (EGM-2; Lonza, Basel, Switzerland) supplemented with 1% penicillin-streptomycin-fungizone (Gibco, Waltham, MA). Cells were cultured at 37 °C and 5% CO_2_. ECs were used from passages four to eight in all experiments. For live cell time-lapse imaging, lentiviral transduction of lifeAct-GFP was utilized.

### Network Formation on Matrigel

Growth factor reduced Matrigel (Corning, Corning, NY) was thawed overnight on ice at 4 °C. 100 μL of thawed Matrigel was pipetted onto 25 mm glutaraldehyde-functionalized glass coverslips and seeded at 4.5 × 10^4^ cells cm^−2^. Coverslips were prepared through exposure to oxygen plasma and subsequent 2 hour incubations in 0.1 mg ml^−1^ poly-L-lysine (PLL) and 5% (v/v) glutaraldehyde. Gelation of Matrigel was completed by incubation at 37 °C for 30 minutes. For variable thickness Matrigel, 25 mm coverslips were first glutaraldehyde-treated as described above. Separate 18 mm coverslips were silanized with trichloro(1H,1H,2H,2H-perfluorooctyl)silane. A small 600 μm thick rectangle of poly(dimethylsiloxane) (PDMS) (Sylgard 184, Dow-Corning, Midland, MI) was then placed on the edge of the glutaraldehyde-treated coverslip, and the silanized coverslip was placed at an angle on the PDMS wedge. 100 μL of Matrigel was slowly pipetted between the two coverslips, incubated at 37 °C for 30 minutes and incubated overnight in PBS. The next day, the silanized coverslip was carefully removed, seeded, and cultured for 12 hours before fixing, staining, and imaging. For studies in which Fl-μS were tracked over time, 0.1% (v/v) blue carboxylate-modified FluoSpheres (1.0 μm diameter, 2% w/v; Life Technologies, Eugene, OR) were added to Matrigel before gelation. For Matrigel crosslinking studies, following gelation, glutaraldehyde solutions of various concentration were pipetted onto the gel and incubated for two minutes and thirty seconds. Samples were then washed twice with 1.0% (w/v) glycine in PBS and incubated for at least 6 hours to neutralize any excess glutaraldehyde in the sample. To quench glutaraldehyde autofluorescence before imaging, substrates were incubated in 1.0% (w/v) sodium borohydride in PBS for 30 minutes at room temperature. To lyse cells on Matrigel, a solution of 20 mM ammonium hydroxide (NH_4_OH) in 0.05% (v/v) Triton-X in PBS was added to the sample, incubated for 5 minutes, and washed with PBS.

### DexMA synthesis

Dextran (MW 86,000 Da, MP Biomedicals, Santa Ana, CA) was methacrylated by reaction with glycidyl methacrylate as previously described^59^. Briefly, 20 mg of dextran and 2 mg of 4-dimethylaminopyridine was dissolved in 100 mL of anhydrous dimethylsulfoxide (DMSO) under vigorous stirring for 12 h. 24.6 mL of glycidyl methacrylate was then added and the reaction mixture was heated to 45 °C for 24 h. The solution was cooled at 4 °C for 1 hour and precipitated into 1 L ice-cold 2-isopropanol. The crude product was recovered by centrifugation, redissolved in milli-Q water, and dialyzed against milli-Q water for 3 days. The final product was lyophilized and stored at −20 °C until use. DexMA was characterized by H-NMR. The degree of functionalization was calculated as the ratio of the averaged methacrylate proton integral (6.174 ppm and 5.713 ppm in D2O) and the anomeric proton of the glycopyranosyl ring (5.166 ppm and 4.923 ppm). As the signal of the anomeric proton of α-1,3 linkages (5.166 ppm) partially overlaps with other protons, a pre-determined ratio of 4% α-1,3 linkages was assumed and the total anomeric proton integral was calculated solely on the basis of the integral at 4.923 ppm. A methacrylate/dextran repeat unit ratio of 0.7 was determined.

### Fiber matrix fabrication

Suspended DexMA fiber matrices were fabricated through electrospinning and soft lithography as previously described^25^. DexMA was dissolved at 0.5 g ml^−1^ in a 1:1 mixture of milli-Q water and dimethylformamide with 0.005% Irgacure 2959 photocrosslinker and 0.002% methacrylated rhodamine (Polysciences, Inc., Warrington, PA). For bead tracking studies, 3.0% (v/v) blue carboxylate-modified FluoSpheres (1.0 μm diameter, 2% w/v) was also added. Electrospinning was completed with a set-up consisting of a high-voltage power supply, syringe pump, and a grounded copper collecting surface enclosed within an environmental chamber at 30% relative humidity. Electrospinning was performed at a flow rate of 0.5 ml h^−1^, voltage of 7.0 kV, and gap distance of 6 cm. Fiber density was varied through modulating electrospinning time and humidity. Samples were primary crosslinked under ultraviolet light to stabilize fibers, hydrated in varying concentrations of lithium phenyl-2,4,6-trimethylbenzoylphosphinate (LAP; Colorado Photopolymer Solutions, Boulder, CO) solution, and then exposed to ultraviolet light (100 mW cm^−2^) for 20 s for secondary crosslinking. LAP concentration was varied between 0.01 and 1.0 mg ml^−1^ to achieve fibers of varying stiffness. Fibers were collected on PDMS arrays of circular wells (2 mm diameter) functionalized with methacrylates to promote fiber adhesion. Briefly, silicon wafer masters possessing SU-8 photoresist (Microchem, Westborough, MA) were produced by standard photolithography and used to generate PDMS stamps. Following silanization with trichloro(1H,1H,2H,2H-perfluorooctyl)silane, stamps were used to emboss uncured PDMS onto oxygen plasma-treated coverslips. Well arrays were methacrylated with vapor-phase silanization of 3-(trimethoxysilyl)propyl methacrylate (Gelest, Inc., Morrisville, PA) in a vacuum oven at 60 °C for at least 6 h. To promote fiber-fiber welding, fiber networks were exposed to a humidified environment for 45 seconds before secondary crosslinking.

### Mechanical testing

To determine the Young’s modulus of Matrigel substrates assuming a linear elastic material, compression testing with a rigid cylinder was performed on a commercial CellScale Microsquisher (CellScale, Waterloo, Ontario). Cylinders (1 mm diameter, 0.5 mm tall) of SU8 photoresist were microfabricated and affixed to pure tungsten filaments (0.156 mm diameter, 59.6 mm length). Matrigel substrates were generated with a height of 450 µm, and indented to a depth of 150 µm at a strain rate of 0.44% s^−1^. Young’s modulus was then calculated as the slope of the linear region (0.04–0.08 strain) of the engineering stress vs. strain plot to remove the influence of a tow region resulting from surface engagement. Young’s moduli of suspended DexMA fiber matrices were measured by microindentation with identical indenters as describe above. Samples were indented to a depth of up to 200 µm at an indentation speed of 2 μm s^−1^. As previously described^25^, Young’s modulus was approximated assuming the material behaves as an elastic membrane using the following equation:

$$F=\frac{Et\pi {\delta }^{3}({r}_{o}^{2}-{r}_{i}^{2})}{2{({r}_{o}-{r}_{i})}^{4}(1-{\rm{\nu }})}$$

where t is the membrane thickness (10 μm, as determined by confocal microscopy), r_o_ is the membrane radius (1 mm), r_i_ is the indenter radius (0.5 mm), ν is the Poisson ratio (0.5), F is the indentation force, δ is the indentation depth, and E is Young’s modulus.

### RGD functionalization and seeding on DexMA fibers

DexMA fibers were functionalized with the cell-adhesive peptide CGRGDS (RGD; Peptides International, Louisville, KY). An RGD concentration of 4 mM was used for all studies. RGD was coupled to available methacrylates via Michael-type addition. Briefly, the peptide was dissolved in milli-Q water containing HEPES (50 mM), phenol red (10 μg ml^−1^), and 1 M NaOH to adjust the pH to 8.0. 200 μL of this solution was added to each substrate and incubated for 30 minutes at room temperature. Following RGD functionalization, substrates were rinsed 2x with PBS before cell seeding. For network formation studies on DexMA fibers, ECs were trypsinized, resuspended in 1.5% (w/v) methylcellulose supplemented EGM-2 to increase media viscosity, and seeded at 6 × 10^4^ cells cm^−2^.

### Pharmacologic contractility inhibition

Blebbistatin, Y-27632, and Cytochalasin D (Santa Cruz Biotechnology, Dallas, TX) were diluted to stock concentrations and stored following the manufacturer’s recommendation. Blebbistatin was utilized at 50 µM in DMSO, Y-27632 at 30 µM in milli-Q water, and Cytochalasin D at 1 µM in DMSO, and samples were treated with pharmacologics at the point of seeding.

### VE-cadherin disruption

EGTA was used to chelate calcium ions, as VE-cadherin engagement at cell-cell adhesions is calcium dependent. Formed EC networks were incubated with 5 mM EGTA at 37 °C for 30 minutes and immediately fixed and processed for fluorescent imaging.

### Bead displacement quantification

For bead displacement analysis to quantify matrix reorganization, time-lapse imaging on a Zeiss LSM 800 confocal microscope (Zeiss, Oberkochen, Germany) was performed, imaging at minimum every 10 minutes for 12 hours. Images were converted to maximum intensity projections, and single particle tracking was completed with TrackMate, a freely available ImageJ plugin^36^, and custom Matlab scripts. Beads were detected at each time point using a difference of Gaussians (DoG) detector with an estimated particle diameter of 5 μm and threshold of 1.0 with use of a median filter. Single particle tracking was completed using a linear assignment problem tracker with a linking max distance and gap-closing max distance of 50 μm and gap-closing max frame gap of 5 frames. Tracks were filtered to only contain particles detected throughout the entire time-lapse, and total displacement for each particle was calculated via custom Matlab scripts.

### Fluorescent staining and microscopy

ECs on Matrigel and DexMA fibers were first fixed in 4% paraformaldehyde for 10 min at room temperature. To stabilize the fibers for long term storage, DexMA samples were crosslinked in 2 mL LAP solution (1.0% w/v) and exposed to UV light (100 mW cm^−2^) for 30 seconds. To stain the actin cytoskeleton and nuclei, cells were permeabilized in PBS solution containing Triton X-100 (5% v/v), sucrose (10% w/v), and magnesium chloride (0.6% w/v), blocked in 1% bovine serum albumin, and stained simultaneously with phalloidin and DAPI. For fibronectin and VE-cadherin immunostaining, samples were fixed and permeabilized (cells were not permeabilized for fibronectin stain) as explained above, blocked for 1 h in 10% fetal bovine serum, and incubated with fibronectin antibody (1:2000, Sigma #F6140) or VE-cadherin (F-8) Alexa-Fluor 488 primary antibody (1:500, Santa Cruz Biotechnology, Dallas, TX) for 1 h at room temperature. Fixed samples and time-lapse microscopy were imaged on a Zeiss LSM 800 laser scanning confocal microscope. Unless otherwise specified, images are presented as maximum intensity projections. Cell area/perimeter and pore analyses were performed using custom Matlab scripts.

### Statistics

Statistical significance was determined by one-way analysis of variance (ANOVA) with post-hoc analysis (Tukey test) or Student’s t-test where appropriate, with significance indicated by p < 0.05. Sample size is indicated within corresponding figure legends and all data are presented as mean ± standard deviation.

## Supplementary information

Movie S1

Movie S2

Movie S3

Movie S4

Movie S5

Supplemental Information

## Data Availability

The datasets generated during and/or analyzed during the current study are available from the corresponding author on reasonable request.
