# Supplementary material for: Cell force-mediated matrix reorganization underlies multicellular network assembly
Source: Sci Rep. 2019 Jan 9;9:12. doi: 10.1038/s41598-018-37044-1 (PMC6327038; doi:10.1038/s41598-018-37044-1)
Supplement: Supplementary file 6 — Supplemental Information [file 41598_2018_37044_MOESM6_ESM.docx]

## Supplemental Information

## Cell force-mediated matrix reorganization underlies multicellular network assembly

Christopher D. Davidson^1^, William Y. Wang^1^, Ina Zaimi^1^, Danica Kristen P. Jayco^1^, Brendon M. Baker^1,+^

^1^ Department of Biomedical Engineering

University of Michigan

Ann Arbor, MI 48109

^+^ Corresponding Author:

Brendon M. Baker, Ph.D.

Assistant Professor, Department of Biomedical Engineering, University of Michigan

2174 Lurie BME Building, 1101 Beal Avenue

Ann Arbor, MI 48109

Email: bambren@umich.edu

## SUPPLEMENTAL FIGURES


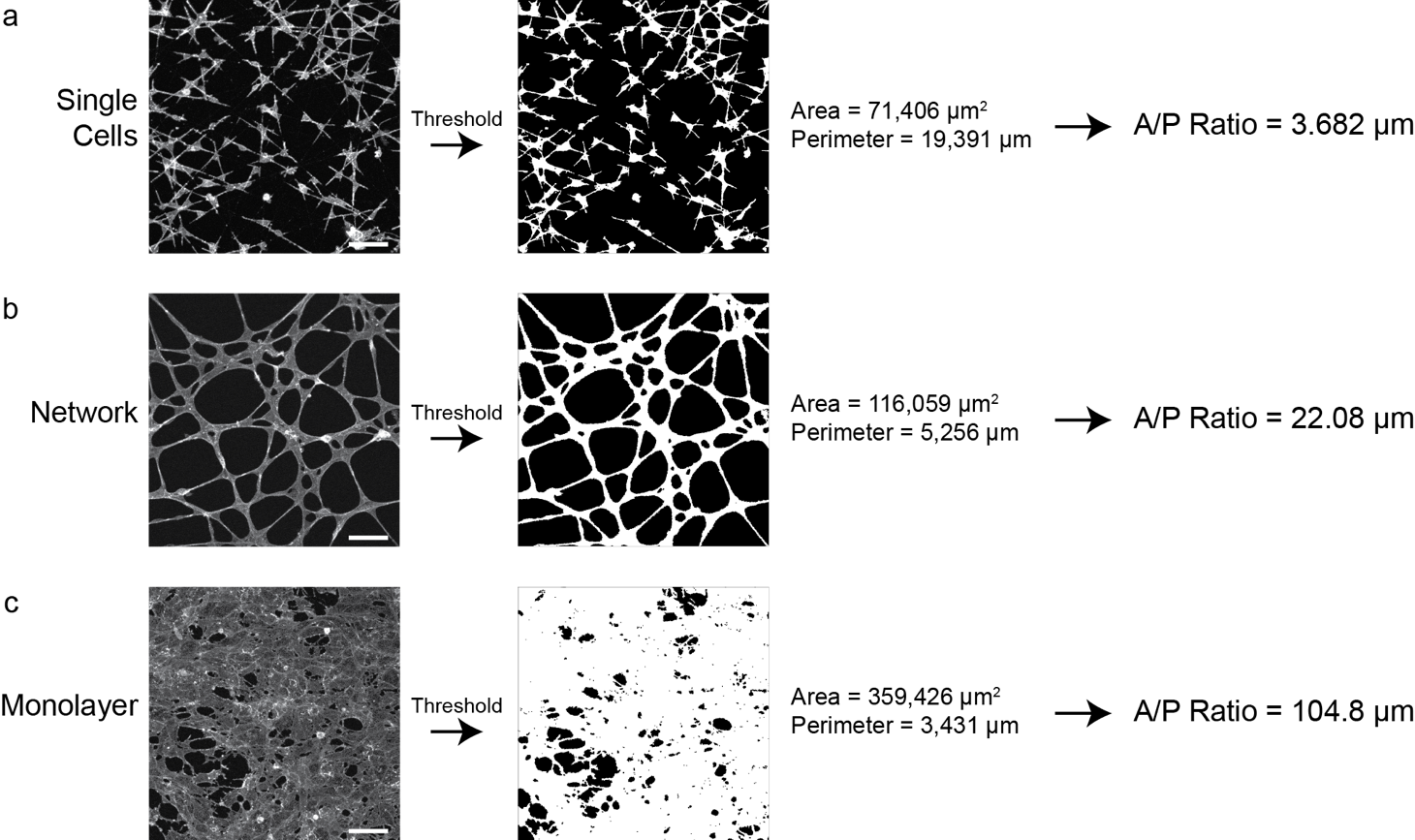


**Figure S1: A/P ratio quantification for three possible cellular phenotypes**

Area/perimeter ratio was quantified by calculating the total area and perimeter of thresholded fluorescent images of phalloidin-stained ECs via custom Matlab scripts. a) Single cells that are not interconnected have a small total area and large perimeter, leading to a relatively low area/perimeter ratio value (between 0 and 10). b) Interconnected cellular networks have an intermediate total area and perimeter, leading to an intermediate area/perimeter ratio value (between 10 and 30). c) Monolayers have a large total area and small perimeter, leading to a relatively high area/perimeter ratio value (greater than 30). Scale bars: 100 μm.

**
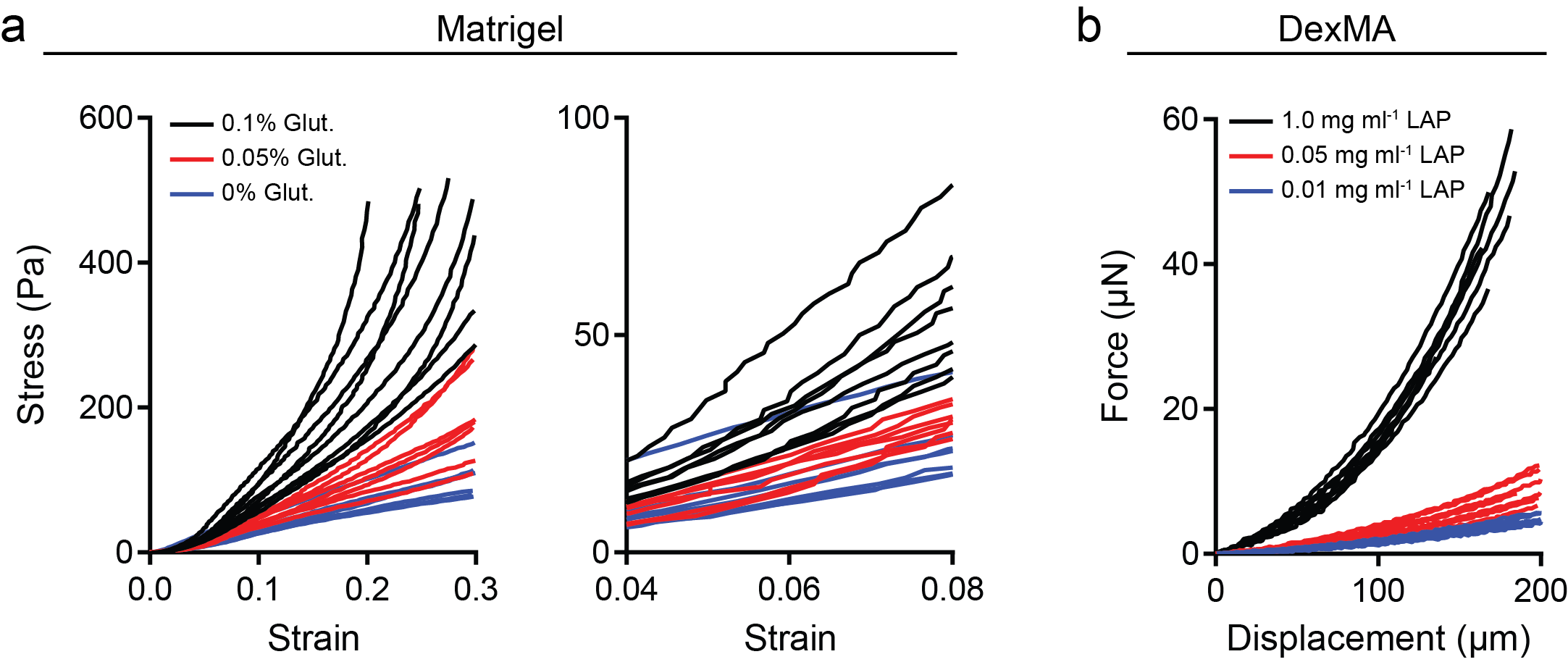
**

**Figure S2: Mechanical characterization of Matrigel and DexMA fibrous matrices.**

a) Stress-strain curves of Matrigel crosslinked with variable concentrations of glutaraldehyde. Right plot shows linear region used to quantify Young’s modulus for each condition. b) Force response of DexMA fibrous matrices as a function of indentation depth of networks crosslinked with varying concentrations of LAP photoinitiator.


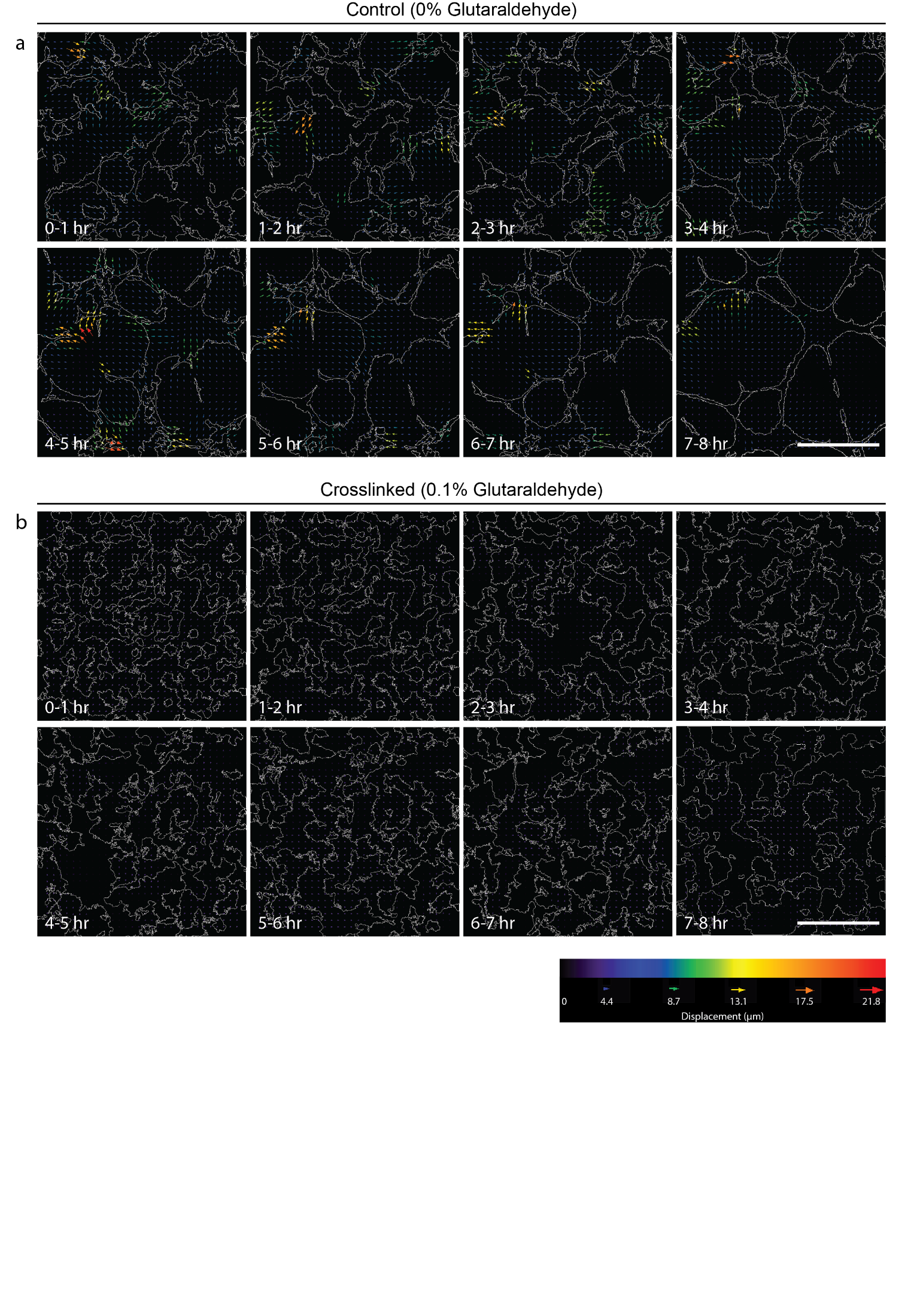


**Figure S3: Matrix reorganization during EC network formation on Matrigel**

Vector plots depict Fl-μS motion with respective EC structure outlined in white for individual 1 hour increments on a) untreated control substrates demonstrating high levels of matrix deformation and organization in contrast to b) glutaraldehyde crosslinked Matrigel. Scale bars: 500 μm.

**
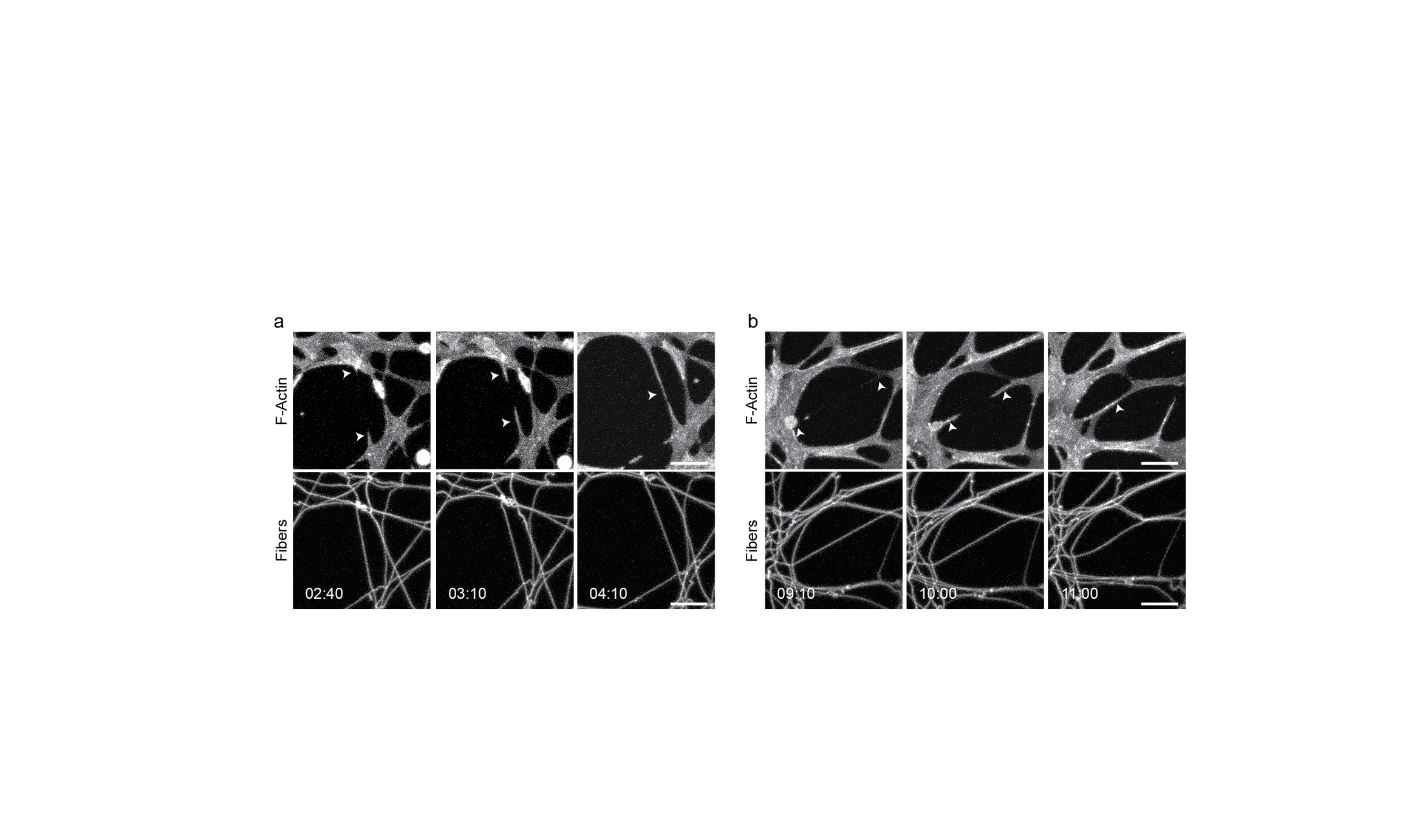
**

**Figure S4: Cellular extensions and inter-connections occur throughout network formation**

Confocal fluorescent images of ECs and rhodamine-labeled fibers showing cellular extensions and the formation of cell-cell interconnections during (a) the first four hours of network formation and (b) after the first four hours of network formation, during network stabilization. Scale bars: 50 μm.


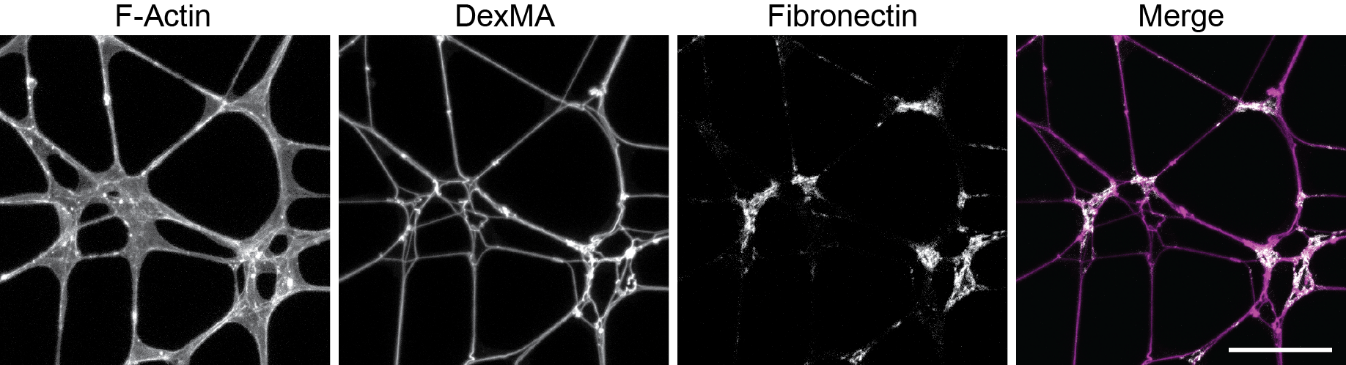


**Figure S5: Fibronectin secretion during EC network formation on fibrous DexMA matrices**

Confocal fluorescent images of ECs, rhodamine-labeled fibers (magenta), and fibronectin (grayscale) after 24 hours of culture on control matrices. Fibronectin secretion was noted at network nodes corresponding to locations with a high relative fiber density due to cell-mediated matrix reorganization. Scale bar: 100 μm.

**
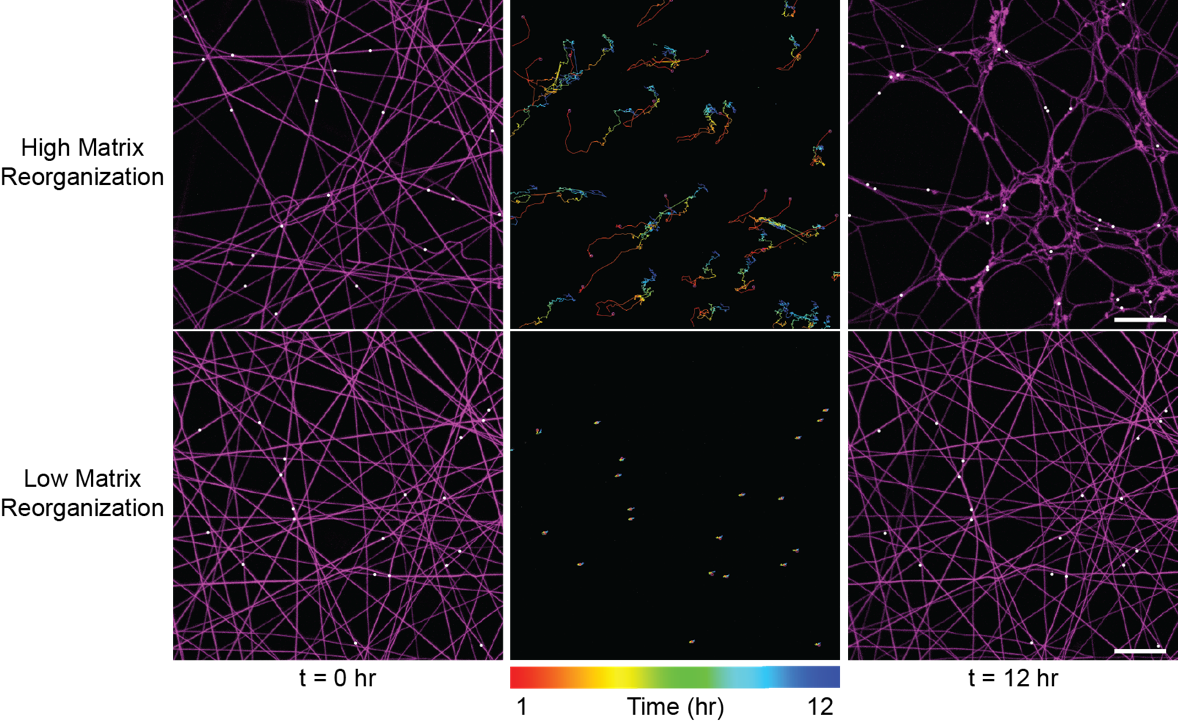
**

**Figure S6: Quantification of Fl-µS displacement using TrackMate**

Matrix reorganization was quantified by calculating the sum of the mean squared displacement of Fl-μS (white) embedded in rhodamine-labeled DexMA fibers (magenta) over 12 hours using TrackMate, an ImageJ plugin ^36^. Conditions that allow for high levels of matrix reorganization demonstrate large displacements, with rapid Fl-μS movement over the first four hours. Conditions with low matrix reorganization exhibit limited Fl-μS displacement. Scale bars: 100 μm.


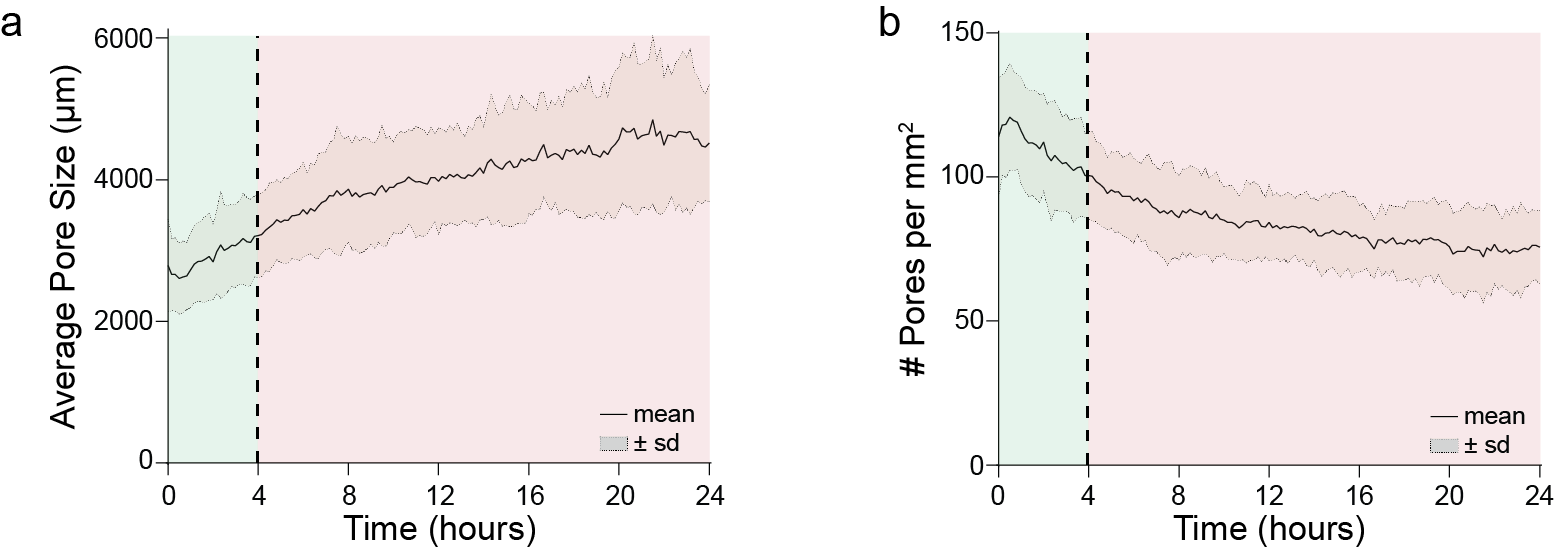


**Figure S7: Quantification of matrix remodeling via analysis of pores during EC network formation on fibrous DexMA matrices.**

Quantitative analysis of pores within DexMA fiber matrices over a 24-hour time-lapse series of network formation. a) Average pore size increases and b) total number of pores decreases during the formation of an EC network, supporting the observation that ECs bundle and condense matrix fibrils as they spread and interconnect; n ≥ 8.


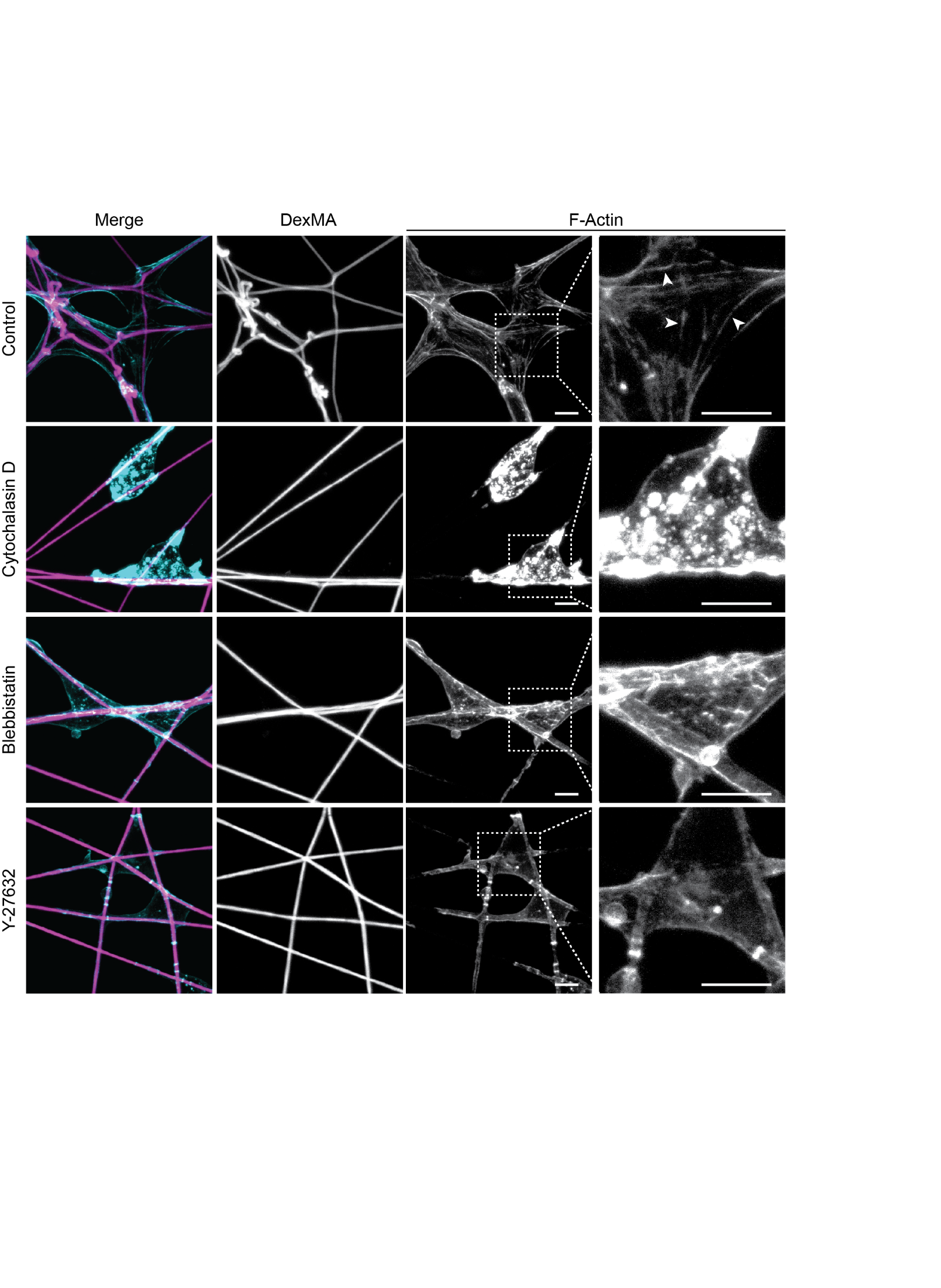


**Figure S8: Analysis of cell cytoskeletal structure after treatment with pharmacologic inhibitors of actomyosin contractility**

High resolution (40x) confocal fluorescence images of phalloidin-stained ECs and rhodamine-labeled fibers after 24 hours of culture with the indicated pharmacologic inhibitor treatment; actin (cyan), fibers (magenta). Dashed boxes indicate locations of higher magnification images depicting variable cytoskeletal structure between different conditions (far right). Scale bars: 10 µm.

**
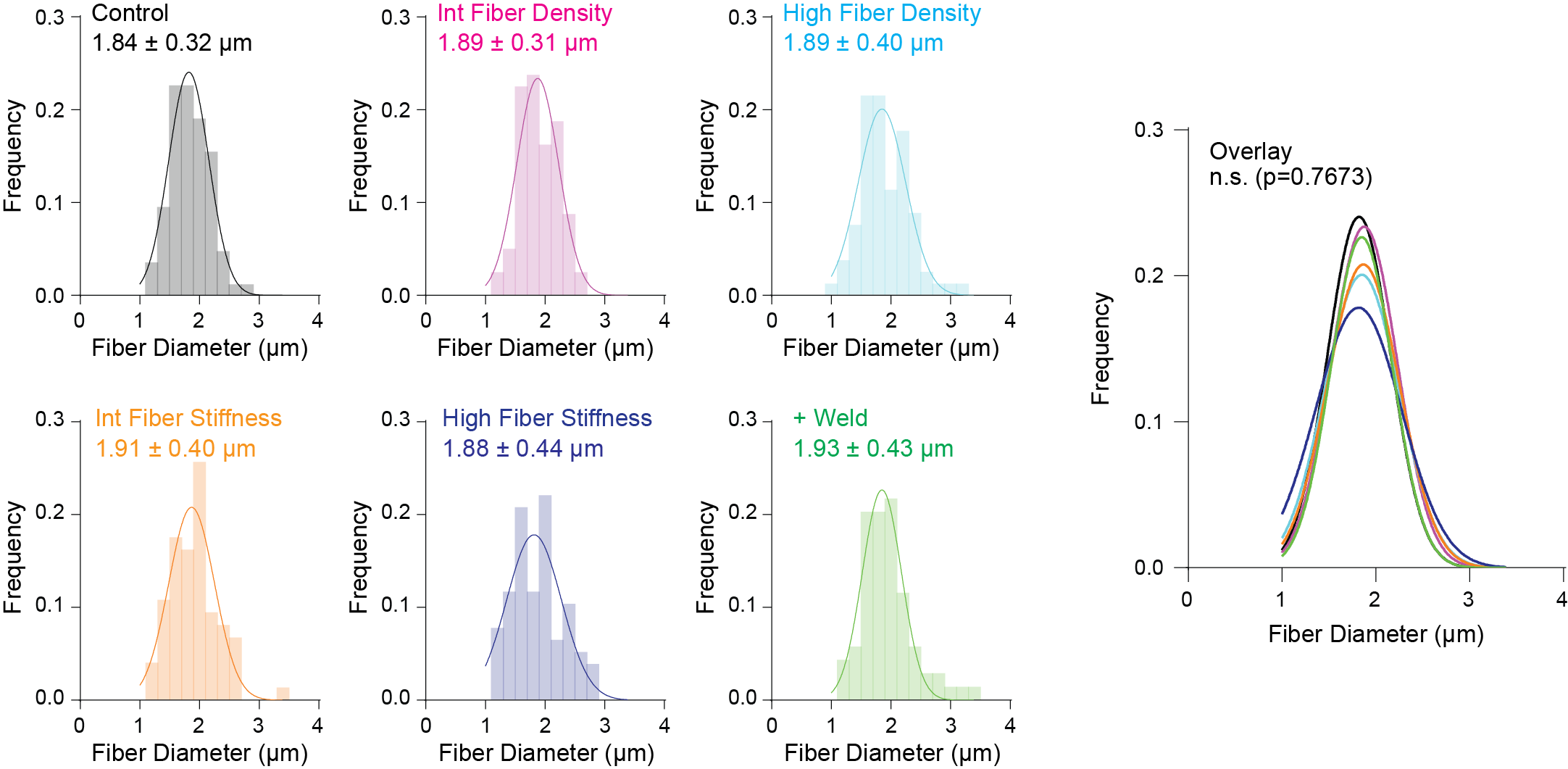
**

**Figure S9: Fiber diameter as a function of physical matrix perturbations**

Histograms showing the distribution of diameter across a population of DexMA fibers with indicated matrix perturbations: control (black), intermediate fiber density (magenta), high fiber density (cyan), intermediate fiber stiffness (orange), high fiber stiffness (purple), and inter-fiber crosslinking via welding (green). For each condition, diameters of n ≥ 69 fibers were quantified; fitted curves assume a Gaussian distribution. Overlay of fitted curves show no statistically significant difference in mean diameter across any matrix perturbation as determined by ANOVA (p=0.7673).

**
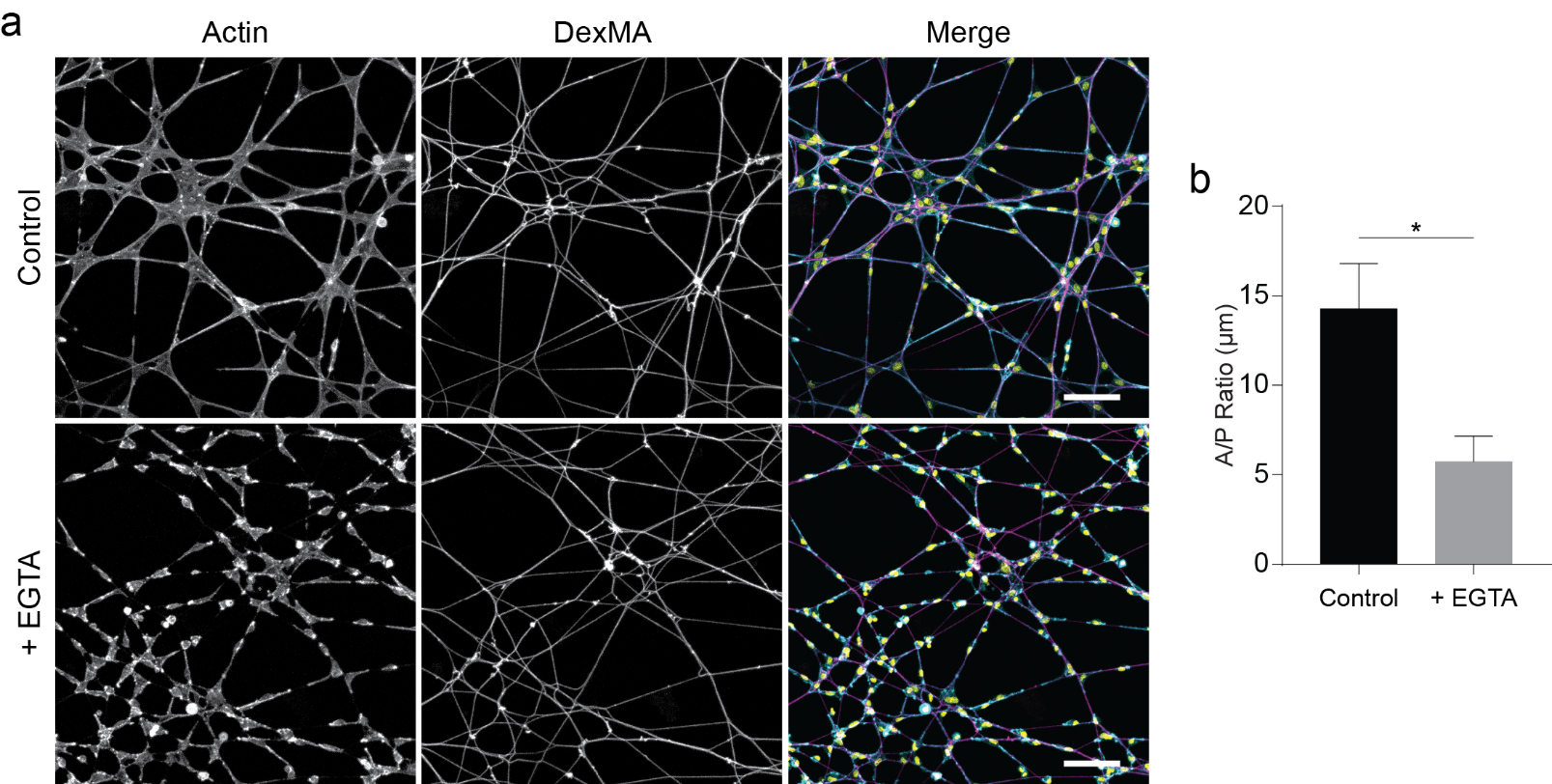
**

**Figure S10: VE-cadherin disruption via calcium chelation alters network architecture**

a) Representative confocal fluorescent images of phalloidin-stained ECs and rhodamine-labeled fibers with and without EGTA treatment to chelate free calcium ions and disrupt cadherin-rich cell-cell adhesions; actin (cyan), fibers (magenta), and nuclei (yellow). (b) Cell area/perimeter ratio after 24 hours of culture and EGTA treatment. Scale bars: 100 μm. Data presented as mean ± std; n = 6; * P<0.05.


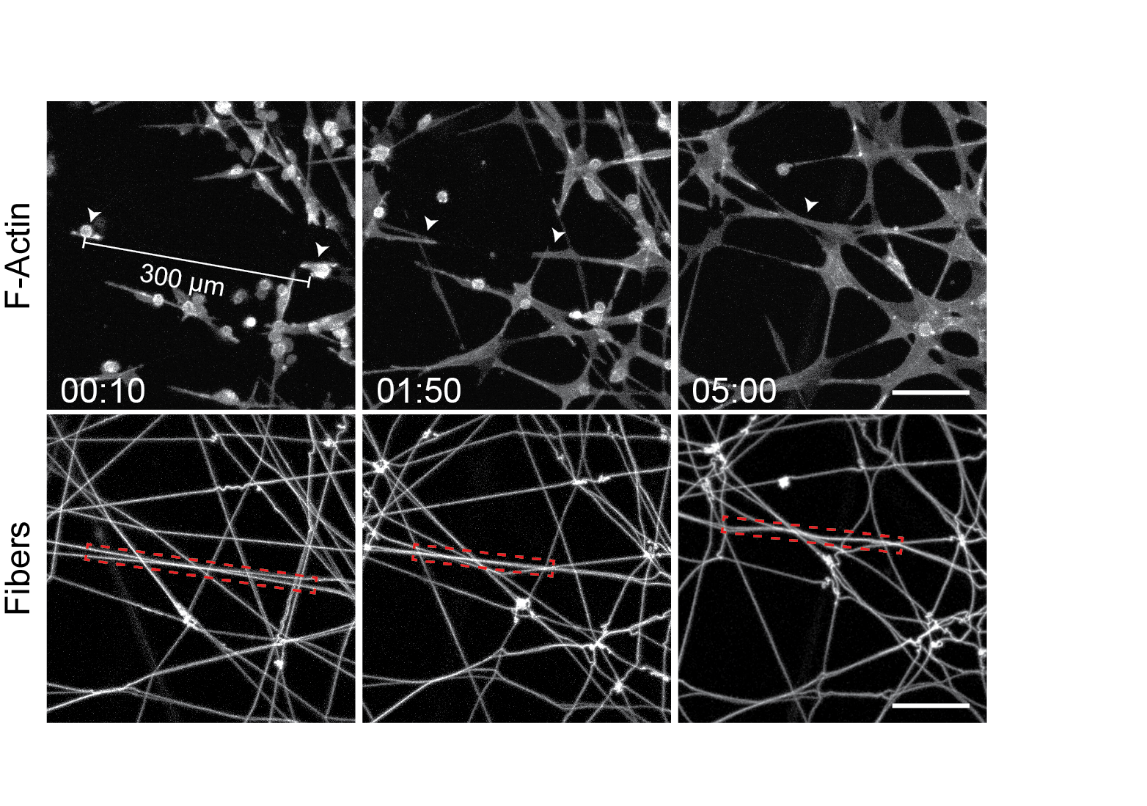


**Figure S11: Long-range cellular extensions during network formation**

Representative confocal fluorescent images of ECs and rhodamine-labeled fibers depicting long-range interactions between cells positioned approximately 300 microns apart. Scale bars: 100 μm.

## SUPPLEMENTAL MOVIE CAPTIONS

**Movie S1: EC network formation and matrix reorganization on Matrigel**

Representative confocal fluorescence time-lapse movie of EC (cyan) network formation on Matrigel with embedded Fl-μS (white). Scale bar: 250 μm.

**Movie S2: EC monolayer formation on glutaraldehyde-crosslinked Matrigel with limited matrix reorganization**

Representative confocal fluorescence time-lapse movie of ECs (cyan) on 0.1% glutaraldehyde crosslinked Matrigel with embedded Fl-μS (white). Scale bar: 250 μm.

**Movie S3: EC network formation and matrix reorganization on synthetic fibrous DexMA matrices**

Representative confocal fluorescence 24-hour time-lapse movie of EC (cyan) network formation on soft (0.01 mg ml^-1^ LAP; 1.5 kPa) synthetic DexMA fiber matrices (magenta). Scale bar: 100 μm.

**Movie S4: Matrix recruitment and cell spreading during first four hours of network formation on DexMA matrices**

Representative confocal fluorescence time-lapse movie of the first four hours of EC (cyan) network formation on soft (0.01 mg ml^-1^ LAP; 1.5 kPa) synthetic DexMA fiber matrices (magenta). Scale bar: 100 μm.

**Movie S5: EC monolayer formation on stiff fibrous DexMA matrices with negligible matrix reorganization**

Representative confocal fluorescence 12-hour time-lapse movie of ECs (cyan) on stiff (0.1 mg ml^-1^ LAP; 16.7 kPa) synthetic DexMA fiber matrices (magenta). Scale bar: 100 μm.
